# Glycolysis Is Required for LPS-Induced Activation and Adhesion of Human CD14^+^CD16^−^ Monocytes

**DOI:** 10.3389/fimmu.2019.02054

**Published:** 2019-09-06

**Authors:** Man K. S. Lee, Annas Al-Sharea, Waled A. Shihata, Camilla Bertuzzo Veiga, Olivia D. Cooney, Andrew J. Fleetwood, Michelle C. Flynn, Ellen Claeson, Clovis S. Palmer, Graeme I. Lancaster, Darren C. Henstridge, John A. Hamilton, Andrew J. Murphy

**Affiliations:** ^1^Division of Immunometabolism, Baker Heart and Diabetes Institute, Melbourne, VIC, Australia; ^2^Department of Diabetes, Monash University, Melbourne, VIC, Australia; ^3^Department of Medicine, The Royal Melbourne Hospital, Parkville, VIC, Australia; ^4^Australian Institute of Musculoskeletal Science, University of Melbourne and Western Health, St. Albans, VIC, Australia; ^5^Faculty of Medicine and Health Sciences, Linköping University, Linköping, Sweden; ^6^Department of Infectious Disease, Burnet Institute, Melbourne, VIC, Australia

**Keywords:** glycolysis, monocytes, inflammation, metabolism, adhesion

## Abstract

Monocytes in humans consist of 3 subsets; CD14^+^CD16^−^ (classical), CD14^+^CD16^+^ (intermediate) and CD14^dim^CD16^+^ (non-classical), which exhibit distinct and heterogeneous responses to activation. During acute inflammation CD14^+^CD16^−^ monocytes are significantly elevated and migrate to the sites of injury via the adhesion cascade. The field of immunometabolism has begun to elucidate the importance of the engagement of specific metabolic pathways in immune cell function. Yet, little is known about monocyte metabolism and the role of metabolism in mediating monocyte activation and adherence to vessels. Accordingly, we aimed to determine whether manipulating the metabolism of CD14^+^CD16^−^ monocytes alters their ability to become activated and adhere. We discovered that LPS stimulation increased the rate of glycolysis in human CD14^+^CD16^−^ monocytes. Inhibition of glycolysis with 2-deoxy-D-glucose blunted LPS-induced activation and adhesion of monocytes. Mechanistically, we found that increased glycolysis was regulated by mTOR-induced glucose transporter (GLUT)-1. Furthermore, enhanced glycolysis increased accumulation of reactive oxygen species (ROS) and activation of p38 MAPK, which lead to activation and adhesion of monocytes. These findings reveal that glycolytic metabolism is critical for the activation of CD14^+^CD16^−^ monocytes and contributes to our understanding of the interplay between metabolic substrate preference and immune cell function.

## Introduction

Innate immune cells such as monocytes play an essential role during inflammation. Monocytes emerge from the bone marrow or spleen into the blood when inflammatory cues are released from sites of injury and ultimately migrate from the blood into the inflamed tissue. This is commonly referred to as the leukocyte adhesion cascade (1). This is a stepwise process initially involving the activation of monocytes, allowing them to roll and tether roll and tether along the activated endothelium followed by firm adhesion and extravasation into inflamed sites. These events are necessary for the survival of the host, as inflammation is important for clearing invading pathogens and repairing damaged tissues (2). On the other hand, excessive inflammation can lead to detrimental effects, resulting in further damage to tissues, and the development of chronic inflammatory diseases such as atherosclerosis and other auto-inflammatory disorders (3–5). Therefore, understanding the underlying mechanisms that regulate monocyte adhesion can provide information on how to manipulate the cell's ability to adhere during acute or chronic inflammation.

It has become increasingly appreciated that the metabolic status of a cell can dictate its functional phenotype. We and others have shown that stimulating cells with inflammatory stimuli switches the energy profile of macrophages and T cells, i.e., from using mitochondrial oxidative phosphorylation (OXPHOS) to glycolysis (6–8). Changes in the cellular substrates and metabolic preferences under resting and activated states has sparked investigations into further understanding immune cell metabolism (i.e., immunometabolism) in relation to their function. It has become increasingly evident that manipulating metabolic pathways influences the development and function of cells. For example, inhibiting glycolysis in LPS-stimulated bone marrow-derived macrophages (BMDMs), prevents the release of the inflammatory cytokine interleukin (IL)-1β (9).

In humans, blood monocytes consist of 3 functionally distinct subsets, CD14^+^CD16^−^ (classical), CD14^+^CD16^+^ (intermediate), and CD14^dim^CD16^−^ (non-classical) (10). CD14^+^CD16^−^ monocytes represent 85% of the circulating monocyte population whereas the other two subsets each make up ~5–8% of the population. In mice, however, monocytes consist of only two subsets, Ly6-C^hi^ and Ly6-C^lo^. Ly6-C^hi^ monocytes are considered to be inflammatory and have been likened to the CD14^+^CD16^−^ monocyte population in humans. During acute inflammation, such as a bacterial infection, these are the subsets that respond first. Therefore, in this study we aimed to investigate how human CD14^+^CD16^−^ monocytes metabolically respond to inflammatory stimuli such as lipopolysaccharide (LPS) and how manipulating monocyte metabolism could alter their functional responses, particularly in relation to their activation and ability to undergo firm adhesion.

## Materials and Methods

### Isolation of Peripheral Blood Mononuclear Cells From Human Buffy Coats

Peripheral blood mononuclear cells (PBMC) were isolated from buffy coats of healthy volunteers supplied by the Australian Red Cross Blood Service via density-centrifugation using Ficoll-Paque solution (density = 1.77). Ethics was obtained through the Alfred Hospital human ethics committee.

### CD14^+^CD16^−^ Monocyte Isolation From PBMCs

PBMCs were resuspended using PBS without Ca^2+^ and Mg^2+^ containing 2 mM EDTA and 5% FBS (FACS buffer) and labeled with a cocktail of fluorescent markers (1:400) consisting of Lin (PE—CD2, PE—CD15, PE—CD56, PE—Nkp46, PE−19), APC—HLA-DR, PB—CD14, PE/Cy7—CD16. After 30 min of incubation on ice, they were then washed and Lin^−^HLA-DR^+^CD14^+^CD16^−^ monocytes were collected via FACS using the BD Aria 1 (Biosciences) at the AMREP Flow cytometry core facility.

### Stimulation/Inhibition of Cells

PBMCs (for flow cytometry assays) and isolated CD14^+^CD16^−^ monocytes were stimulated with lipopolysaccharides (LPS) (100 ng/ml) for 1 h in the presence or absence of 1 h of metabolic inhibitor pre-treatment; glycolysis inhibitor: 2-Deoxy-D Glucose (2DG) (5 mM), mechanistic target of rapamycin (mTOR) inhibitor: rapamycin (20 nM), reactive oxygen species (ROS) inhibitor: NAC (1 mM), p38 MAPK inhibitor: SB-203580 (5 nM) and mitochondrial ROS scavenger: MitoQ (100 nM).

### Flow Cytometry

PBMCs were resuspended in 200 μl of FACs buffer. A cocktail of the fluorophores (1:400) were added to stain for the different monocyte subsets. These consisted of Lin (PE—CD2, PE—CD15, PE—CD56, PE—Nkp46, PE−19), APC—HLA-DR, PB—CD14, PE/Cy7—CD16. To measure CD11b levels or GLUT-1 expression, FITC- CD11b (1:400), and FITC—GLUT-1 was also added, respectively. After incubating on ice for 30 min, they were then washed and transferred into FACS tubes. In order to measure metabolism of cells via flow cytometry, 10 nM MitoTracker Deep Red, 4 μM 2-NBDG, 5 μM MitoSOX, and 10 μM H2DCFDA were stained in RPMI 1640 and incubated in 37°C for 20 min before they were washed and transferred into FACS tubes. Cells were immediately run on the BD LSRII Fortessa (BD Biosciences). 100,000 cells were collected for analysis. Unstained and single stained controls were used to set up voltages to compensate for spectral overlap. Flow cytometry data were quantified using FlowJo vX0.7 (FlowJo LCC) software.

### Seahorse Bioanalyser Assay

Monocytes were pre-treated with inhibitors and seeded at 100,000 cells/well in the XF^e^ 96 well cell culture microplate (Agilent Technologies). The microplate was spun at 1,000 RPM for 5 min at 4°C; acc = 5, dec = 0 to obtain a monolayer of monocytes in each well. The supernatant was discarded and 175 μl of seahorse media [XF based minimal DMEM (Agilent Technologies) supplemented with 5.5 mM Glucose Solution (Gibco), 1 mM Sodium Pyruvate (Gibco) and 2 mM L-Glutamine (Gibco)], containing the same concentrations of inhibitors, were carefully added so as not to disturb the cell layer. The XF^e^ 96 well cell culture microplate was incubated at 37°C in a non-CO_2_ incubator for at least 30 min. The assay cartridge was hydrated overnight with 200 μl of XF Calibrant Media (Agilent Technologies) at 37°C (in non-CO_2_ incubator) before LPS was suspended in 25 μl of seahorse media and was added to Port A of the assay cartridge at a concentration of 100 ng/ml. Basal extracellular acidification rate (ECAR) was measured for 4 × 6.5 min cycles. LPS was automatically injected into the XF^e^ cell culture plate after the 4th cycle and ECAR readings were recorded for 1 h post-LPS injection.

### Vessel Chamber Adhesion Assay

Aortic vessels were isolated from C57BL/6 mice and stimulated in Krebs buffer with bovine serum albumin (BSA) (1:1,000 w/v) and TNF-α for 4 h at 37°C. TNF-α activated vessels were mounted onto the cannulas and Krebs solution warmed at 37°C was used to flood the vessel chamber to mimic *in-vivo* conditions. Monocytes were re-suspended in 6 ml of RPMI at the concentration of 1 × 10^6^ cells/ml. 1 mM of Vybrant Dil (Invitrogen) was added for 10 min in dark conditions, to fluorescently label the cells. Cell solutions were transferred into a terafusion syringe pump (Teruma) which was used to direct the movement of the cells through the aortic vessel at a rate of 7.1 ml per hour. Images of adhered monocytes were taken using the Zesiss Discover V.20 Fluorescence Microscope (Carl Zeiss MicroImaging) mounted on a Hammastsu HD Camera (Hamamatsu®) at 0, 2.5, 5, 7.5, and 10 min. Data was then quantified by calculating the number of stationary fluorescent dots per field of view (FOV).

### F-Actin Assay

Eight well chamber slides (Lab-Tek) were pre-coated with 200 μl/well of fibrinogen (100 μg/ml) and incubated overnight at 4°C. The next day, each well was washed twice with PBS without Mg^2+^ and Ca^2+^ to get rid of non-adhered fibrinogen via aspiration. Two-hundred microliter of 3% BSA were then added and incubated at room temperature for 15 minutes before washing again with PBS twice. Two-hundred microliter of monocytes (at 1 × 10^6^/ml in RPMI 1640 media) were then added into each well and stimulated with 100 ng/ml LPS for 1 h at 37°C. Cells were then washed with PBS twice to remove unbound monocytes. Two-hundred microliter of 4% para-formaldehyde (PFA) was then added for 15 min at room temperature to fix adhered monocytes onto slides. Again, PBS was used to wash off the PFA before permeabilising cells with 0.1% Triton X-100 for 10 min at room temperature. After washing cells with PBS, 200 μl of PBS containing 10% FBS was used to block any non-specific binding for 15 min at room temperature. Cells were washed again before staining with fluorescent markers of F-actin (33 nM phalloidin) and nucleus (1 ng/ml 4′,6-diamidino-2-phenylindole (DAPI) for 20 min in the dark at room temperature. Cells were then washed twice with PBS and the gasket were removed. slides were finally allowed to completely dry before mounting on No. 2 glass coverslips (Menzel) using Dako fluorescence mounting media. Imaging was performed through the monash micro imaging core, on a Nikon A1r confocal microscope using NIS-elements software (Nikon) at 60X magnification. To quantify F-actin staining, the fluorescence intensity of phalloidin stain per cell, normalized to cell size, was measured using Image J. Moreover, cells were counted individually, using the Image J count function, to quantify the number of adherent monocytes.

### Western Blot

Protein samples were isolated from lysed Monocytes. A 10% SDS-PAGE gel was used to separate the protein samples which were subsequently transferred onto a nitrocellulose membrane. Five percent fat-free skim milk in tris-buffered saline with tween (TBST) was used to block the membrane for non-specific binding and washed with before the addition of various primary antibodies (1:1,000) consisting of p-mTOR (Santa Cruz), p-ERK1/2 (Cell Signaling Technologies), β-actin (Cell Signaling Technologies), p-p38 MAPK (Cell Signaling Technologies), p38 MAPK (Cell Signaling Technologies), and HSP90 (Cell Signaling Technologies). Membranes were then incubated overnight at 4°C. Appropriate secondary antibodies (1:2,000) were added for 1 h at room temperature and subsequently washed before visualization of the protein bands using enhanced chemi-luminescence reagents (PerkinElmer) and quantified using Quantity One (Bio-Rad) software.

### Statistical Analyses

Data are presented as mean ± SEM where each individual donor was denoted by n. *P* values were calculated by using unpaired Student's *t*-test or one-way ANOVA followed by Tukey's *post-hoc* test using Graphpad Prism 7 (Graphpad Software). *P-*values of <0.05 were deemed to be statistically significant.

## Results

### LPS Activation Increases Glycolysis in Human CD14^+^CD16^−^ Monocytes

**Figure 1:**
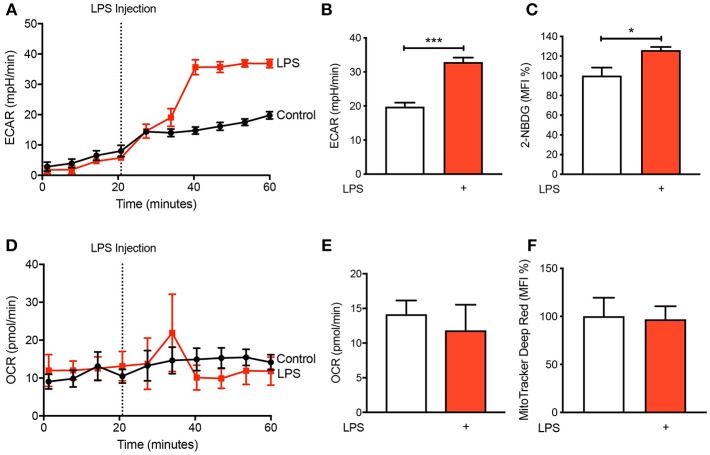
LPS increases glycolysis in human CD14^+^CD16^−^ monocytes. Isolated human CD14^+^CD16^−^ monocytes were treated with or without 100 ng/ml LPS. A seahorse bioanalyser was used to measure extracellular acidification rate (ECAR) **(A,B)**; *n* = 4. Flow cytometry was used to measure glucose uptake using fluorescent analog 2-NBDG **(C)**; *n* = 6. A seahorse bioanalyser was used to measure oxygen consumption rate (OCR) **(D,E)**; *n* = 4. Mitochondrial activity was measured using flow cytometry **(F)**; *n* = 7. Data are mean ± SEM (un-paired *t*-test: *p < 0.05, ***p < 0.001).

It is becoming increasingly appreciated that immune cells alter their metabolism when they become activated (11, 12). To determine the metabolic preference of human CD14^+^CD16^−^ monocytes after activation with LPS, we isolated cells from buffy coats of healthy volunteers via fluorescence-activated cell sorting (FACS) before placing them in the seahorse XF^e^ 96 bioanalyser to characterize their metabolic preference. We noted a significant increase in extracellular acidification rate (ECAR) within 20 min of LPS stimulation (Figures 1A,B) indicating increased rate of glycolysis. This was also associated with enhanced glucose uptake, measured via flow cytometry using 2-NBDG (Figure 1C). Moreover, we found no significant change in oxygen consumption rate (OCR), which is a proxy of oxidative phosphorylation (OXPHOS), in CD14^+^CD16^−^ monocytes after LPS stimulation (Figures 1D,E). We also used flow cytometry to quantify mitochondrial activity, which confirmed our findings from the seahorse bioanalyser (Figure 1F). These data suggest that stimulating monocytes with LPS causes an increase in glycolysis while not affecting OXPHOS.

### Inhibiting Glycolysis Decreases Monocyte Activation and Adhesion

**Figure 2:**
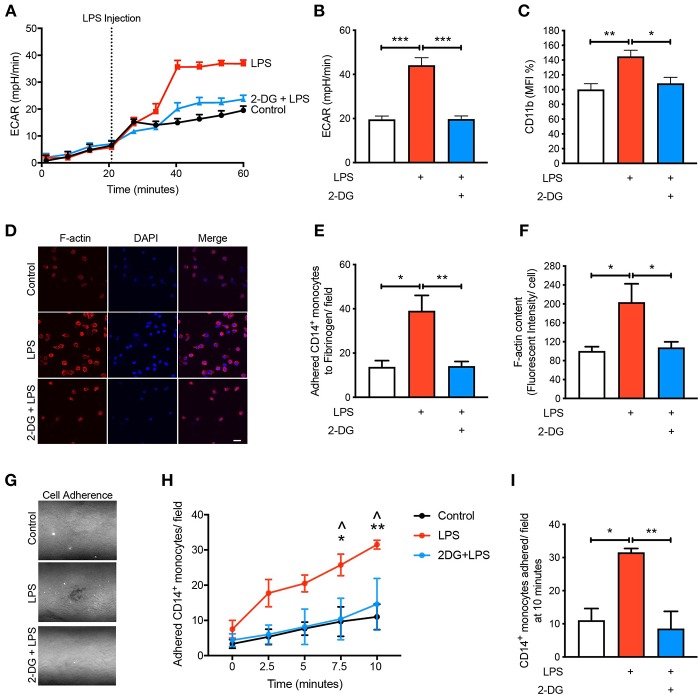
Glycolysis is required for LPS-induced monocyte activation and adhesion. Isolated human CD14^+^CD16^−^ monocytes were pre-treated with or without 5 mM 2-DG for 1 h followed by 1 h of 100 ng/ml LPS stimulation. Seahorse bioanalyser was used to measure extracellular acidification rate (ECAR) **(A,B)**; *n* = 4. Flow cytometry was used to measure CD11b expression **(C)**; *n* = 6–8. Representative images (20 μm scale bar) **(D)** of the number of adhered monocytes **(E)** and F-actin content measured via confocal microscopy **(F)**; *n* = 3–5. Representative image of shear flow adhesion assay (white dots = adhered cells) **(G)**. Quantification over 10 min time course **(H)** and at 10 min **(I)**; *n* = 3–5. Data are mean ± SEM (one-way ANOVA with Tukey's test: *&^∧^*p* < 0.05, **&*p* < 0.01, ***p < 0.0001). In **(H)**, ^∧^ denotes comparison between LPS vs. 2DG-LPS.

Given that we found CD14^+^CD16^−^ monocytes increase glycolysis following LPS stimulation, we aimed to determine how important glucose utilization was to their ability to activate in response to LPS. To do this we used 2-Deoxy-D-glucose (2-DG), a glucose analog that enters into the cell like glucose but inhibits the first step of glycolysis via competitively blocking hexokinase, a rate limiting step of glycolysis (13). As expected, pre-treating cells with 2-DG reduced the increase in glycolysis caused by LPS (Figures 2A,B). Additionally, pre-treating cells with 2-DG significantly inhibited the activation of the CD14^+^CD16^−^ monocytes as determined by the cell surface activation marker CD11b (Figure 2C). To functionally confirm the role of glycolysis in LPS-induced monocyte adhesion, we performed a static adhesion assay where we pre-coated wells with fibrinogen which allows activated monocytes to bind via CD11b. Monocyte adhesion was assessed using confocal microscopy where cells were also stained to quantify F-actin content as another measure of cell activation. As expected, following LPS stimulation, there was a significant increase in the number of monocytes adhering to fibrinogen as well as an increase in F-actin content. These effects were blunted when cells were pre-treated with 2-DG (Figures 2D–F). Furthermore, we performed a shear flow cell adhesion assay to monitor in real-time monocyte adhesion under shear stress in TNF-α activated mouse aorta *ex vivo*. We treated CD14^+^CD16^−^ human monocytes with LPS 1 h before flowing the cells through the endothelium and found that there was a significant increase in monocyte adhesion. This increase in monocyte adhesion was abolished when pre-treating with 2-DG (Figures 2G–I). This confirms our hypothesis that blocking glycolysis prevents LPS-induced monocyte activation and adhesion.

### mTOR Is Involved in Regulating Glycolysis in LPS-Induced Monocytes

**Figure 3:**
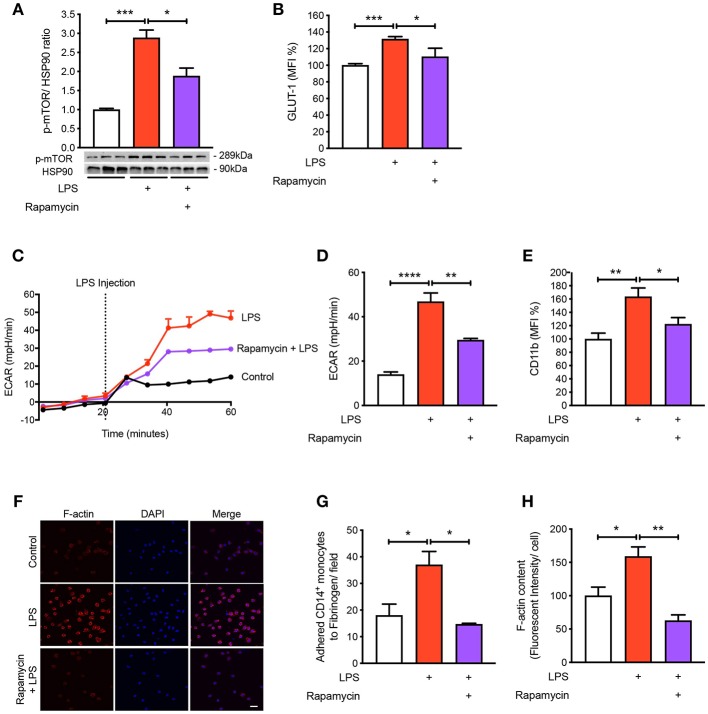
mTOR pathway is involved in regulating glycolysis in LPS-induced monocytes. Isolated human CD14^+^CD16^−^ monocytes were pre-treated with or without 20 nM rapamycin an hour before 1 h 100 ng/ml LPS stimulation. mTOR phosphorylation was quantified by western blot at 30 min after LPS stimulation **(A)**; *n* = 6–7. Flow cytometry was used to measure GLUT-1 expression; *n* = 3–5 **(B)**. Extracellular acidification rate (ECAR) was measured in real-time **(C,D)**; *n* = 3–4. CD11b expression was measured by flow cytometry **(E)**; *n* = 6–7. Static cell adhesion assay performed utilizing F-actin and DAPI stain via confocal microscopy (20 μm scale bar) **(F–H)**; *n* = 3–4. Data are mean ± SEM (un-paired *t*-test and one-way ANOVA with Tukey's test: *p < 0.05, **p < 0.01, ***p < 0.001, ****p < 0.0001).

To understand the mechanisms by which LPS increases glycolysis, we explored the mTOR pathway as it has been known to be involved in regulating glycolysis (14). Following LPS treatment we found a significant increase in the phosphorylation of mTOR in the CD14^+^CD16^−^ monocytes and using rapamycin as a positive control for mTOR phosphorylation, phosphorylation of mTOR was significantly reduced as expected (Figure 3A). The activation of mTOR is also known to further upregulate glucose transporter (GLUT)-1 to the cell surface in order to facilitate increased glucose uptake (15). When we measured GLUT-1 expression using flow cytometry, we found that LPS significantly increased cell surface GLUT-1 levels. When we inhibited mTOR activity using rapamycin, GLUT-1 expression was significantly blunted in LPS-induced monocytes (Figure 3B). This suggests that the increase in cell surface GLUT-1 is regulated by mTOR in monocytes. Furthermore, we also showed that inhibiting mTOR significantly prevented the increase in LPS-stimulated glycolysis (Figures 3C,D), suggesting mTOR is a master regulator of glycolysis in CD14^+^CD16^−^ monocytes. Next, we blocked mTOR to determine whether this affected the activation and adhesion of monocytes. When we pre-treated cells with rapamycin, we were able to prevent CD11b expression in LPS-induced monocytes (Figure 3E). The anti-inflammatory effects of mTOR blockade were also seen in the static adhesion assay where pre-treating cells with rapamycin prevented the number of monocytes adhered to fibrinogen. In addition, we found a decrease in F-actin content (Figures 3F–H). This suggests that the mTOR pathway is involved in LPS-induced activation and adhesion of CD14^+^CD16^−^ monocytes by controlling glucose uptake and glycolysis.

### Blocking p-38 MAPK Signaling Prevents LPS-Induced Activation and Adhesion

**Figure 4:**
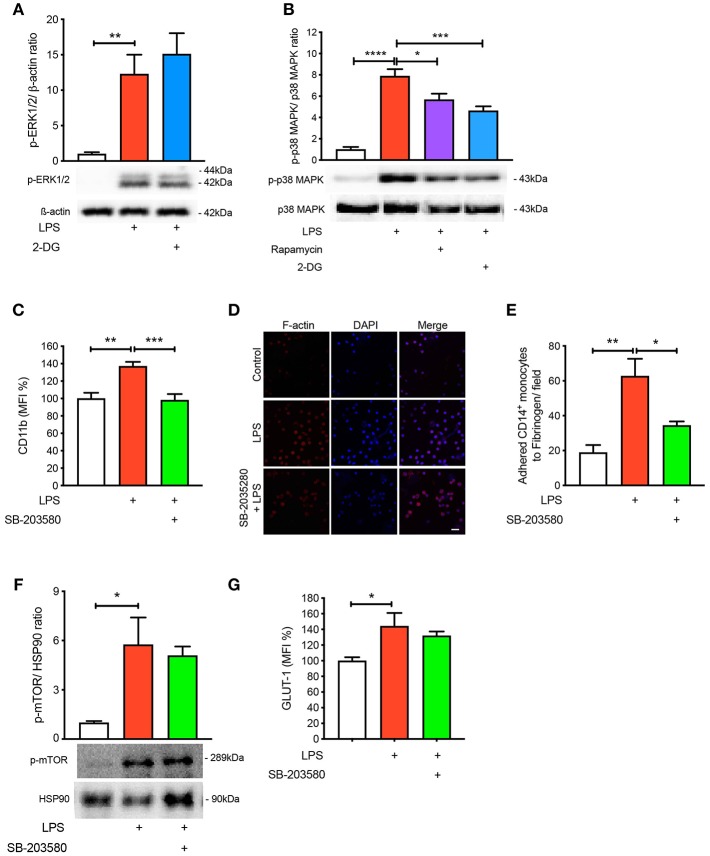
p38 MAPK is involved in LPS-induced monocyte activation and adhesion. ERK1/2 phosphorylation were measured with or without 5 mM 2-DG followed by 1 h of 100 ng/ml LPS stimulation **(A)**; *n* = 4–5. p38 MAPK phosphorylation was measured with or without 5 mM 2-DG or 20 nM rapamycin before 100 ng/ml LPS stimulation **(B)**; *n* = 6–7. Cells were pre-treated with 5 nM SB-203580 followed by 1 h of 100 ng/ml LPS stimulation before measuring CD11b expression via flow cytometry **(C)**; *n* = 8. Static cell adhesion assay was performed utilizing F-actin and DAPI stain via confocal microscopy (20 μm scale bar); *n* = 3–4 **(D–E)**. mTOR phosphorylation was quantified by western blot at 30 min after LPS stimulation in the presence of 5 nM SB-203580 **(F)**; *n* = 3. Flow cytometry was used to measure GLUT-1 expression; *n* = 3–4 **(G)**. Data are mean ± SEM (one-way ANOVA with Tukey's test: *p < 0.05, **p < 0.01, ***p < 0.001, ****p < 0.0001).

To further understand the signaling pathways mediating the activation and adhesion of LPS-stimulated monocytes, we measured mitogen-activated protein kinases (MAPKs), extracellular signal-related kinases (ERKs) and p38 MAPK, which are known to be activated during inflammation (16–18). As expected, both MAPKs were significantly phosphorylated when monocytes were treated with LPS (Figures 4A,B). Interestingly, blocking glycolysis using 2-DG did not prevent the phosphorylation of ERK1/2 (Figure 4A). Moreover, when we pre-treated cells with rapamycin and 2-DG, p38 MAPK phosphorylation was significantly decreased (Figure 4B). Therefore, we decided to inhibit p38 MAPK to determine whether this affected LPS-induced monocyte activation and adhesion. We employed SB-203580, an inhibitor of p38 MAPK, which confirmed that this pathway was involved in monocyte activation as we noted LPS was no longer able to increase the cell surface expression of CD11b (Figure 4C). Consistent with the inability of LPS to induce CD11b, we also found a reduction in the number of monocytes adhering to fibrinogen when p38 MAPK was inhibited (Figures 4D,E). To further confirm p38 MAPK occurs downstream of mTOR and glycolysis, we measured mTOR phosphorylation via western blot and also GLUT-1 levels via flow cytometry with SB-203580 in the presence of LPS and confirmed that phosphorylation of mTOR and GLUT-1 levels were unchanged (Figures 4F,G). These data suggest that LPS-mediated glycolysis and mTOR signaling induce p38 MAPK to promote CD14^+^CD16^−^ monocyte activation and adhesion.

### Reactive Oxygen Species Are Involved in Glycolysis-Mediated Activation and Adhesion

**Figure 5:**
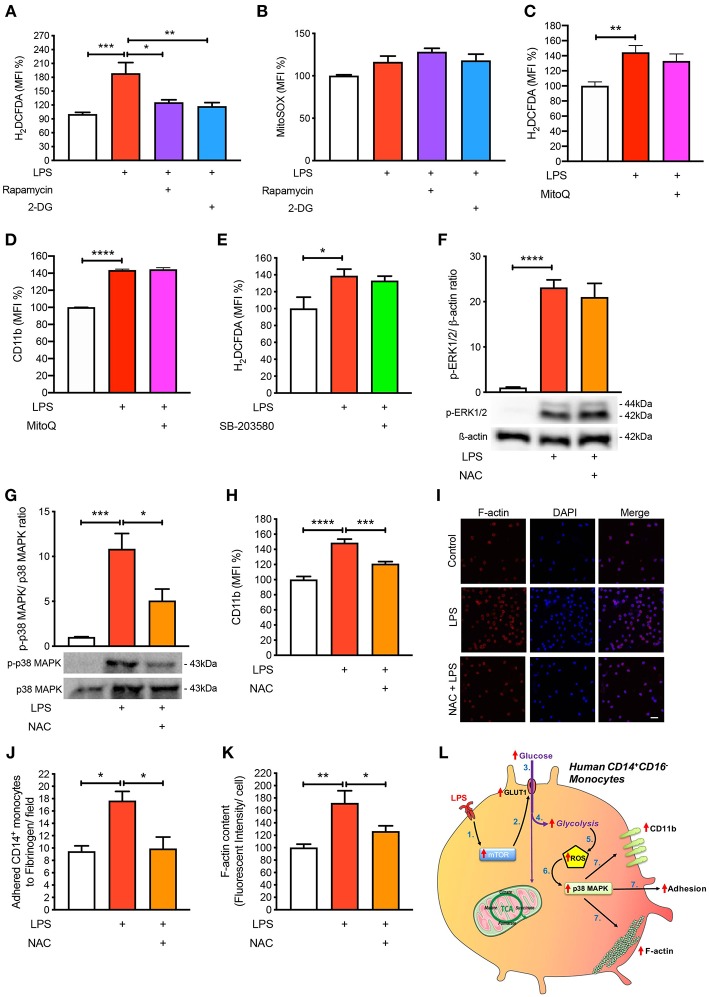
ROS regulates p38 MAPK-induced monocyte activation and adhesion. Isolated human CD14^+^CD16^−^ monocytes were pre-treated with or without 20 nM rapamycin or 5 mM 2-DG followed by 1 h of 100 ng/ml LPS stimulation before H_2_DCFDA **(A)** and MitoSOX **(B)** levels were measured via flow cytometry; *n* = 4–8. Cells were pre-treated with 100 nM MitoQ followed by 1 h of 100 ng/ml LPS stimulation before H_2_DCFDA **(C)** and CD11b **(D)** levels were measured via flow cytometry; *n* = 7–8. Cells were pre-treated with 5 nM SB-203580 followed by 1 h of 100 ng/ml LPS stimulation before measuring H_2_DCFDA levels via flow cytometry **(E)**; *n* = 4. Cells were pre-treated with or without 1 mM NAC before stimulating with 100 ng/ml LPS for 30 min before measuring ERK1/2 **(F)** and p38 MAPK **(G)** phosphorylation; *n* = 4–5. CD11b expression was measured via flow cytometry; *n* = 6–8 **(H)**; *n* = 6–7. Static cell adhesion assay performed utilizing F-actin and DAPI stain via confocal microscopy (20 μm scale bar) **(I–K)**; *n* = 3–6. Schematic diagram of proposed mechanistic pathway of acute LPS-induced CD14^+^CD16^+^ monocyte activation and adhesion **(L)**. Data are mean ± SEM (one-way ANOVA with Tukey's test: *p < 0.05, **p < 0.01, ***p < 0.001, ****p < 0.0001).

Next, we aimed to mechanistically link glycolysis with the induction of p38 MAPK signaling in driving CD14^+^CD16^−^ monocyte activation and adhesion. Inflammatory signaling can trigger p38 MAPK activation by ROS and preventing ROS accumulation using anti-oxidants averts p38 MAPK activation (16, 19, 20). Since one of the by-products of glycolysis is ROS generation, we hypothesized that this may be the link between glycolysis and p38 MAPK activation. Indeed, we found that LPS increased ROS accumulation in monocytes via flow cytometry (Figure 5A). Our data suggest that ROS generation was a consequence of increased glycolysis, as interventions upstream of glycolysis, rapamycin, and 2-DG, were able to block global ROS levels using the H_2_DCFDA fluorescent indicator (Figure 5A). Moreover, to determine whether the increase in ROS production was generated from glycolysis and not glucose utilization via the mitochondria, we specifically stained for mitochondrial ROS using MitoSOX, which we found to be unchanged with LPS (Figure 5B). To confirm that mitochondrial ROS did not play a role during acute LPS activation, we treated cells with MitoQ that specifically reduces mitochondrial ROS and showed that global ROS levels (Figure 5C) and CD11b expression were unchanged (Figure 5D). We also measured ROS production in LPS-induced monocytes that were pre-treated with the p38 MAPK inhibitor SB-203580 and found no change in ROS, suggesting that p38 MAPK is downstream of ROS in LPS-activated monocytes (Figure 5E). Next, we inhibited ROS oxidation of cysteines using N-acetyl-L-cysteine (NAC) and measured phosphorylation of p38 MAPK to delineate whether ROS activated p38 MAPK. Indeed, we found that pre-treating cells with NAC before incubating with LPS prevented p38 MAPK activation but not ERK1/2 phosphorylation, suggesting that the ROS driven pathway was specific to p38 MAPK signaling (Figures 5F,G). These results show that ROS production in LPS-induced monocytes occurs downstream of the mTOR and glycolytic pathway but upstream of p38 MAPK. More importantly, inhibiting ROS production using NAC, significantly prevented LPS-induced CD11b expression (Figure 5H). Furthermore, NAC was able to affect LPS-induced monocyte adhesion as well as reduce F-actin formation (Figures 5I–K). These results indicate that LPS stimulation of monocytes triggers mTOR regulated glycolysis, which drives ROS, causing downstream p38 MAPK activation, resulting in monocyte activation and adhesion.

## Discussion

During an acute inflammatory response, monocytes become activated, adhere to the endothelium, and transmigrate into the inflamed tissue. However, the mechanistic pathways by which human CD14^+^CD16^−^ monocytes activate and adhere, in particular the specific metabolic pathways are yet to be fully elucidated. In this study, we revealed that increased flux through glycolysis is critical to induce the signaling pathways that monocytes rely on for adherence. We found that LPS-stimulated human CD14^+^CD16^−^ monocytes increase CD11b expression and adhesion via the phosphorylation of mTOR which facilitates the uptake of glucose and glycolysis. When we further investigated the mechanistic link between glycolysis and adhesion, we found that an increase in glycolysis resulted in the production of ROS which activated the p38 MAPK pathway, leading to monocyte activation and adhesion.

Glycolysis and oxidative phosphorylation (OXPHOS) via the mitochondria are the two main metabolic pathways, which can control the overall phenotype of the cell. A classic example of this are the metabolic status of inflammatory and anti-inflammatory macrophages. Inflammatory or M1-like macrophages are highly glycolytic, while anti-inflammatory or M2-like macrophages have been found to be mitochondrial dependent, using both glucose and fatty acids for OXPHOS (11, 12). More importantly, when glycolysis or OXPHOS are inhibited using specific metabolic inhibitors it reduces the ability of macrophages to become inflammatory or anti-inflammatory, respectively. Here, we demonstrate a similar scenario in human CD14^+^CD16^−^ monocytes where LPS increased glucose uptake and glycolysis. This is consistent with findings from Stienstra's group who also show an increase in glycolysis with LPS (21). However, it was somewhat surprising OXPHOS or mitochondrial activity was not reduced in the presence of LPS. This could suggest that the mitochondria does not play an essential role in providing the increase in energy metabolism for cellular activation in human CD14^+^CD16^−^ monocytes during short (1 h) exposure compared to 24 h of LPS stimulation, where many other cellular changes are likely to be occurring (21). The non-reliance on the mitochondria in acute responses could be because CD14^+^CD16^−^ monocytes do not require much energy as they are carried around the body by the circulatory system and require rapid activation upon an inflammatory stimulus and so have evolved to require little respirative metabolism. Thus, consistent with the hypothesis that CD14^+^CD16^−^ monocytes use glycolytic metabolism, when this pathway was inhibited using 2-DG, we found a loss in the ability of these monocytes to increase cell surface CD11b and to adhere. This builds on a growing body of evidence that the cellular metabolic preference is a key determinate of cellular function.

Previously, it has been noted that mTOR is involved in regulating cell adhesion in cancer cells; however, the mechanistic pathway downstream of mTOR has not been explored. mTOR, the central regulator of cellular growth and proliferation, also governs glycolysis. Studies in BMDMs have shown that mTOR is responsible for upregulating glucose transporters, namely GLUT-1 (15). Glucose transporters are involved in facilitating the uptake of glucose which increases the rate of glycolysis. This pathway was also activated in our human CD14^+^CD16^−^ monocytes as inhibition of mTOR with rapamycin prevented GLUT-1 movement to the surface as well as glycolysis, leading to a reduction in monocytic activation and adhesion.

Delving further into the mechanisms linking glycolysis to the activation and adhesion of LPS-induced monocytes, LPS has previously been known to stimulate many activation pathways including p38 MAPK and ERK1/2 in human CD14^+^CD16^−^ monocytes. Additionally, p38 MAPK and ERK1/2 have been shown to regulate adhesion of tumor-associated macrophages, suggesting their involvement is important in cellular activation in order to cause adhesion to vessels or a matrix (16, 18). Interestingly, only p38 MAPK was found to be important in glycolysis-mediated cell adhesion in the CD14^+^CD16^−^ monocytes, suggesting that glycolytic events are key in regulating the phosphorylation of p38 MAPK. One of the stressors that is capable of signaling via p38 MAPK is ROS. Within the glycolytic pathway, we found that non-mitochondrial generated ROS appeared to activate p38 MAPK in order to cause LPS-induced monocyte activation. The source of non-mitochondrial ROS is likely to be NADPH-oxidase (NOX)-dependent, which is induced via the glycolytic pathway (22). Additionally, studies have shown that NOX enzymes are increased in human monocytes and macrophages upon inflammatory conditions in addition with increased ROS levels (23).

Clinical trials testing the benefits of antioxidants in CVD-related clinical trials have been disappointing, with many larger clinical trials showing no beneficial effects when given antioxidants such as vitamin E, C or coenzyme Q. After careful reflection on these trials, the limitations of these studies should be considered before closing the door to the therapeutic potential of antioxidants in CVD. These include dosage, efficacy, *in vivo* biological activity, specificity and statistical power (24–26). However, the importance of ROS in cellular metabolism in CVD is regaining traction. Importantly, we are now gaining a better understanding on the regulator of cellular ROS and how to harness the power of endogenous antioxidant pathways such as Nrf2 or restoring mitochondrial health (if the mitochondria is the driver) (27). Another important point to consider is the timing of intervention to therapeutic gain, perhaps anti-oxidants need to be administered earlier in life as opposed to testing this in middle to older aged participants in trial setting and formulating appropriate primary endpoints. How glycolysis regulates p38 MAPK is currently unknown, but given p38 MAPK is downstream of ROS, it is likely that MKK3/6, and potentially ASK1, are intermediary targets (19, 28). Nonetheless, LPS-induced glycolysis is required for the phosphorylation of p38 MAPK to cause monocyte activation.

In summary, we have found that the metabolism of glucose by human CD14^+^CD16^−^ monocytes in response to LPS is critical for the activation of these cells (Figure 5L). These findings contribute to a larger body of evidence revealing that changes in cellular metabolism are central for the cell to respond to extrinsic stimuli (bacterial, viral, cytokines, etc). These metabolic changes assist the cell in performing effector functions and as such have become a key interest in disrupting unwanted processes, particularly in the immune system. Understanding these pathways and selectively inhibiting glycolysis may aid in chronic diseases where excessive monocyte recruitment is detrimental.

## Data Availability

The datasets generated for this study are available on request to the corresponding author.

## Author Contributions

ML, AA-S, EC, WS, CB, OC, and MF conducted the experiments and analysis. ML, AA-S, AF, CP, GL, DH, JH, and AM contributed to the experimental design and intellectual input. ML, AA-S, and AM wrote and revised the manuscript. All authors contributed to editing the manuscript.

### Conflict of Interest Statement

The authors declare that the research was conducted in the absence of any commercial or financial relationships that could be construed as a potential conflict of interest. The handling editor declared a past collaboration with the authors ML and AM.
